# The New Year

**Published:** 1883-01

**Authors:** 


### THE NEW YEAR.

It is with undisguised pleasure that we send to the friends of the REGISTER, wherever they may be, our sincere wishes for a happy and prosperous year.

Among all the toil and hardships of the physician's life there are, fortunately, many bright spots and sunny experiences, which serve to cheer and encourage him in his somewhat isolated, often dreary and never-ceasing combat with the foes of poor humanity; and it is our wish that, in this year of grace, these helpful oases along life's journey may be multiplied, and that unstinted and substantial rewards may crown the honest efforts of all worthy workers within the ranks of our honored profession.

It has been said that the rewards within the reach of the general practitioner are few and inferior. But this, it seems to us, depends largely upon the individual. It is true we cannot successfully compete with many callings in amassing wealth; yet in the face of this admitted fact, we hold that in no vocation is there a wider or more inviting field for well directed industry than general medicine affords, and this advantage is admirably and eloquently set forth by Sir James Paget in his address as president of the last International Medical Congress.

In the course of that address he said: "Let us always remember the nobility of our calling. I dare to claim for it, that among all the sciences, ours, in the pursuit and use of truth, offers the most complete and constant union of those three qualities which have the greatest charm for pure and active minds—novelty, utility and charity.

"We can compete with the world in the nobler ambition of being counted among the learned and good, who strive to make the future better and happier than the past. And to this we shall attain if we will remind ourselves that in every pursuit of knowledge there is the charm of novelty, and in every attainment of truth utility, so in every use of it there may be charity. I do not mean only the charity which finds its expression in hospitals or in the service of the poor, great as is the privilege of our calling in that we may be its chief ministers, but that wider charity which is practiced in a constant sympathy and gentleness, in patience and self-devotion And it is surely fair to hold, as in every search for knowlegde we may strengthen our intellectual power, so in every practical employment of it, we may, if we will, improve our moral nature. We may obey the whole law of Christian love, we may illustrate the highest induction of scientific philanthropy. Let us, then, resolve to devote ourselves to the promotion of the whole science, art and charity of medicine."

In this just view of the opportunities and possibilities of our chosen pursuit there is surely encouragement and reward enough to kindle the interest of all real lovers of our science, and to renew the energies of all true disciples of our art. This ought, we say, to satisfy the most exalted and far-reaching ambition.

It would be a source of genuine gratification to feel that our greeting will find all of our readers entering the new year with joyous and hopeful surroundings, but, alas! we feel that it is the fate of our race to share the bitter with the sweet, and too often to garner a harvest of sorrow, when the spring-time gave promise of abundant returns of happiness. Yes,

"There's a minor in the carol,
A shadow in the light,
And a spray of cypress twining
In the holly wreath to-night."

To these we say "Good Cheer," for we know full well that the darkest night ends with the dawn, and that even in the midst of present gloom there is an over-ruling wisdom which directs and controls the affairs of men. The horizon, closely scanned, foretells the coming day. It will not always be dark. Let us learn to labor and to wait; to toil without ceasing; to abide our time in patient well-doing, and we shall, at least, rejoice in the sustaining knowledge that we deserve, even if we fail to achieve, success.

